# Horizontal Plasmonic Ruler Based on the Scattering Far-Field Pattern

**DOI:** 10.3390/s18103365

**Published:** 2018-10-09

**Authors:** Eunso Shin, Young Jin Lee, Youngsoo Kim, Soon-Hong Kwon

**Affiliations:** Department of Physics, Chung-Ang University, Seoul 156-756, Korea; ensoshin21@gmail.com (E.S.); youngjin.lee.91@gmail.com (Y.J.L.); youngsoo.kim94@gmail.com (Y.K.)

**Keywords:** plasmonic ruler, far-field, scattering

## Abstract

A novel method is proposed to detect the horizontal shift of a specific nanoblock relative to a reference nanoblock using surface plasmon modes at nanometer resolution. To accomplish this task, two orthogonal localized surface plasmon resonances were excited within the air gap region between the silver nanoblocks at the respective wavelengths, 890 nm, and 1100 nm. This technique utilized the scattering far-field intensities of the two block nanostructures at the two specific wavelengths at two specific directional spots. The ratio of the scattering intensities at the two spots changed according to the horizontal shift of the block that moved. Correspondingly, this ratio can be used to provide the precise location of the block. This method can be applied to many fields, including label-free bio-sensing, bio-analysis and alignment during nano-fabrication, owing to the high resolution and simplicity of the process.

## 1. Introduction

Remarkable improvements in nanotechnology have led to new prospects in many fields such as electronics, nanophotonics, and the biological sciences. One of the key techniques in nanophotonics is surface plasmon resonance (SPR) [1,2]. SPR is an optical phenomenon that results from strong light-matter interaction, thus leading to resonance oscillation of electron charges excited by the incident light at the interface between two materials with different permittivity values. These different positive and negative permittivity values are mostly associated with dielectric and metallic materials [3,4,5,6,7]. At the resonance wavelength, the amplitude of the near-field is enhanced owing to the strong confinement at the interface. Correspondingly, the scattering light pattern in the far-field is considerably modified by the nanostructure because of the SPR. As the intensity of the confined light is strongly enhanced, the SPR has the ability to manipulate light at the subwavelength length scale, thereby achieving very high spatial resolution.

These technical aspects regarding SPR can be applied to numerous applications, including ultrasensitive detectors for physical and chemical sensing [8,9] relative to the refractive index of the environmental material, gas detection such as hydrogen concentration [10], and spatial position measurement [11,12,13,14,15]. In addition, these SPR sensors can be extensively exploited for biophysical applications [16,17] including the analysis of molecules [18,19,20], their detection and the monitoring of changes in their spatial distribution. A plasmon ruler can measure minute spatial changes of the structure of a molecule by using spectrum analysis of plasmon resonance modes in metal nanostructures [21,22,23,24]. However, measurements of the scattering spectra of the nanostructures, such as nanoparticle and nanoblocks, remains challenging, and the spectral resolution is intrinsically limited by the broad linewidth of the plasmon resonance.

In this investigation, we theoretically propose a simple and sensitive method to detect any relative movement along the horizontal direction between the two metal nanoblocks at a nanometer resolution. Accordingly, this method uses the relative ratio of the scattering intensities in the far-field based on two specific directions for two different plasmonic modes. Even though measurement techniques for near-field optical properties are rapidly developing [25,26,27,28], measurements in the far-field regime are significantly easier to be implemented and are more cost-effective.

## 2. Structure, Specific Mode Profile and Calculation Methods

**Figure 1:**
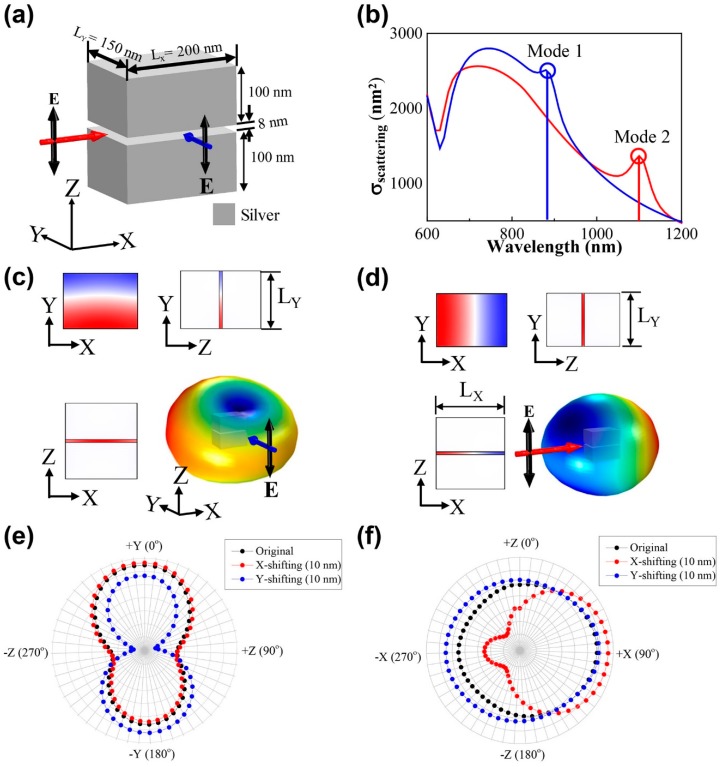
(**a**) Schematic of the two-metal block assembly when light propagates along the X-axis and the Y-axis with the electric field oriented in the vertical direction. (**b**) Scattering far-field spectra of the two-metal block assembly using light propagates along the X-axis and Y-axis. Simulated electric field distribution in the YZ, XZ plane and also XY plane with resonance wavelengths of (**c**) 890 nm and (**d**) 1100 nm. Additionally, the 3D distribution of the far-field with (**c**) Y-directional and (**d**) X-directional incident light propagation. Polar graphs are also plotted to depict the scattering far-field in YZ plane and XZ plane for the wavelengths of (**e**) 890 nm and (**f**) 1100 nm, respectively.

We hereby propose a plasmonic ruler consisting of two silver nanoblocks with an air gap of 8 nm whereby each block has the same geometric parameters, namely, L_X_ = 200 nm, L_Y_ = 150 nm and a height of 100 nm as shown in Figure 1a. Depending on the direction of the incident light, one of the two plasmonic modes can be independently excited in the air gap. To excite the gap modes, light is linearly polarized along the Z-direction (E_Z_). When this light is incident on the nanoblocks along the Y-direction (X-direction), as indicated by the blue (red) arrow in Figure 1a mode 1 (or mode 2) is excited at 890 nm (or at 1100 nm), as observed in the scattering cross-section (σ_sc_) spectrum of Figure 1b. Even though the scattering intensity changes are observed at lower and higher wavelengths, the elicited changes are maximized at the resonant peaks of 890 nm and 1100 nm. Therefore, we chose these two wavelengths as the reference wavelengths.

Figure 1c,d show mode profiles for mode 1 and 2 at the wavelengths of 890 nm and 1100 nm, respectively. In both modes, the electric fields are strongly confined in the air gap between the two blocks, and there exists one intensity node. However, the node of mode 1 is directed along the X-axis in contrast to the Y-axis in the case of mode 2. These two modes can be excited separately depending on the direction of the incident light because of these different mode profiles. In addition, the optical response of each mode is distinct for the X- or Y-directional displacements of the upper block, while the position of the lower block is fixed [13]. The scattering far-field patterns of mode 1 and mode 2 vary in different ways.

**Figure 2:**
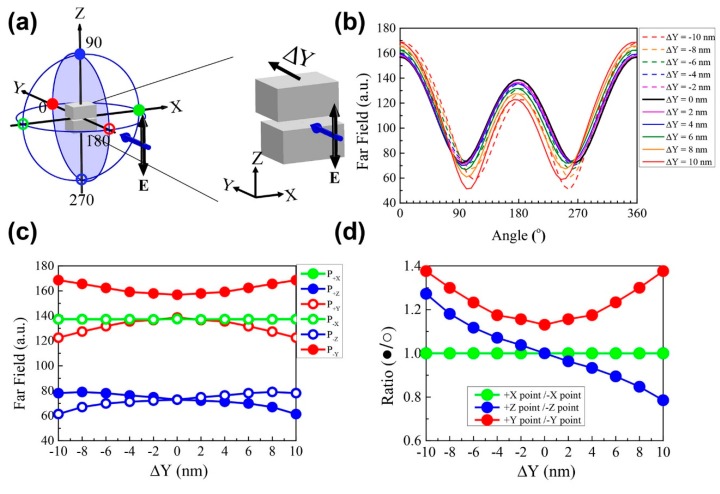
(**a**) Indicated by the blue sphere is the numerical calculation domain of the far-field region in the case of upper block shifts in the +Y direction (right side of subfigure). The blue arrow indicates the incident light propagating along the Y-axis with the vertical electric field (E_Z_), as shown by the black arrow. (**b**) Scattering far field intensity plots in the YZ plane as a function of angle for upper block shifts. (**c**) Plots of far-field values at specific spots along the ±X, ±Y, ±Z directions. All colored dots indicate the respective axes depicted in Figure 2a. (**d**) Plots of scattering intensity ratios obtained by dividing the value at each positive axis location with the corresponding value at each negative axis location in the YZ plane.

Firstly, we calculated the scattering far-field patterns of mode 1 for three different displacements of the upper block: for the original position (black), for an X-shift of 10 nm (red), and for a Y-shift of 10 nm (blue), respectively, as shown in Figure 1e. The simulation was conducted using COMSOL. These shifts were observed from the scattering of incident light at 890 nm. In the polar graphs plotted in the YZ planes, the far-field pattern in the case of a 10 nm shift in the X direction was almost similar to that of the original position of the upper block. However, the far-field pattern for a 10 nm shift in the Y direction resulted in a significant change compared to that of the original position. In contrast, the far-field patterns of mode 2 at 1100 nm showed an opposite dependence on the horizontal displacement of the upper block. The far-field pattern for an X-shift displacement of 10 nm yielded a significant change in contrast to the minor change observed for a Y-shift displacement of 10 in the XZ plane at the far-field. Conversely, mode 1 (or mode 2) yielded similar far-field patterns for the X or Y directional shifts in the XZ (or YZ) planes. These distinguishable dependencies of the scattering far-fields of mode 1 (890 nm) and mode 2 (1100 nm) on the horizontal displacement, enabled the measurement of the horizontal position of the upper block with respect to the lower block by observing the scattering far-fields at 890 nm and 1100 nm. Since the scattering field of mode 1 elicits significant changes for Y directional shifts of the upper block in Figure 1, it is expected that the measurement of the scattering field of mode 1 using an incident light at a wavelength of 890 nm should allow the determination of the Y-position of the upper block to be determined. Therefore, we investigated the scattering fields for incident light that propagated along the Y direction to excite mode 1, as shown in Figure 2a, as the upper block was shifted from −10 nm to +10 nm at a 2 nm step intervals along the Y-axis.

## 3. Novel Method for Measuring 1D Locations

The scattering fields in the YZ plane were calculated as a function of the azimuthal angle in the YZ plane because the change of the scattering field along the X direction was insignificant, as indicated by the scattering intensity. In Figure 2a, the filled-blue circles indicate the YZ plane. In addition, angle values are measured from the +Y-axis (0°) and increases in the clockwise direction so that the angle for +Z-axis is 90°, the angle for −Y-axis is 180°, and the angle for the −Z-axis is 270°. In Figure 2b, the solid lines indicate the scattering intensity for a directional shift of the upper block along the +Y-axis, and the dashed lines correspond to a directional shift of this block along the −Y-axis. The original structure is represented by the black solid line at ΔY = 0 nm, and has the same magnitude of the scattering field magnitude in the +Z (90°) and −Z (270°) directions. However, the scattering fields for +Y-axis (0 °), forward scattering) and −Y-axis (180°, backward scattering) are quite different. 

The solid line indicates that the upper block is shifted along the −Y-axis. Regarding the forward scattering field, more scattering occurs when the upper block is shifted by 10 nm in the +Y-direction (ΔY = 10 nm) compared to the original structure (ΔY = 0 nm), while backward scattering is less than that of the original structure. When the upper block moved by −10 nm (ΔY = −10 nm), the forward and backward scattering fields were same to those elicited at ΔY = +10 nm. Conversely, the scattering fields in the +Z (90 °) and −Z (270 °) directions exhibit opposite trends for ΔY = +10 nm and ΔY = −10 nm. When ΔY = +10 nm, the scattering intensity at +Z is lower than that at −Z. However, the scattering intensity at +Z is higher than that at −Z for a shift of ΔY = −10 nm. There are six dots around each circle which represents each direction. The filled dots show the direction of increasing values along the direction of interest, whereas the hollow dots represent decreasing values. In addition, the green dots represent the direction of the X-axis, while the red dots represent the Y-axis, and the blue dots represent the Z-axis.

Based on the differences of the scattering fields at the ±Y and ±Z directions for Y-shifts of the upper block in the opposite direction, we investigated the scattering intensities as a functions of the Y-shift from −10 nm to +10 nm for six axis directions, namely, ±X, ±Y, ±Z, as shown in Figure 2c. The filled (hollow) dots indicate the scattering intensity along the + (-) axis direction. In the case of the +X and −X directions (green), the scattering intensities are the same as for those for the +X and −X directions, and the value is extending beyond the range of the Y-shift because of the symmetry of the system which contains the Y-shifted blocks. Along the +Y direction (solid red circles), the scattering field intensity increases as the absolute value of the Y-shift increases. In contrast, the scattering field along the −Y-axis (hollow red circles) decreases as the upper block shifts more along the +Y or −Y directions with respect to the origin. However, the scattering field at the ±Y axis elicits the same dependency as a function of the Y-shift, regardless of the ±sign of the shift. Conversely, for the +Z direction (solid blue line), the scattering intensity decreases monotonically from 80 to 60 as the Y-shift of the upper block increases from −10 nm to +10 nm. For the Z direction (hollow blue circles), the scattering intensity shows exactly the opposite tendency, and increases monotonically from 60 to 80. The properties of +Z and−Z directional scatterings are unique compared to the other axial directions. The +X and −X directional scatterings do not depend on distance or direction. However, the +Y and −Y directional scatterings depend on distance but not on direction. The +Z and −Z directional scatterings have distinguishable dependencies with respect to the distance and direction of the Y-shift of the upper block.

In order to use the unique dependencies of the scattering field on the Y-shift in the ±axial direction, we divided the scattering intensity in two components, namely, one along the positive axial direction (solid circles), and another along the negative axial direction (hollow circles), as shown in Figure 2d. As expected in Figure 2c, because the scattering is the same for the +X and −X directions, the ratio of the scattering field (green) was maintained at the value of unity in the range of the Y-shift. The ratio (+Y/−Y) indicated by the red line increased as the upper block shifted more from the origin. However, the plot of the ratio was symmetric in the +Y and −Y directions. Conversely, the ratio of the scattering intensities in the +Z and −Z axial directions monotonically decreased from 1.4 to 0.6 for the Y-shifts of the upper block from −10 nm to +10 nm. Therefore, if the scattering intensities for the incident light of 890 nm were measured in the +Z and –Z directions (mode 1), the ratio of the scattering field could determine the position of the shifted upper block in the Y direction at a nanometer resolution.

**Figure 3:**
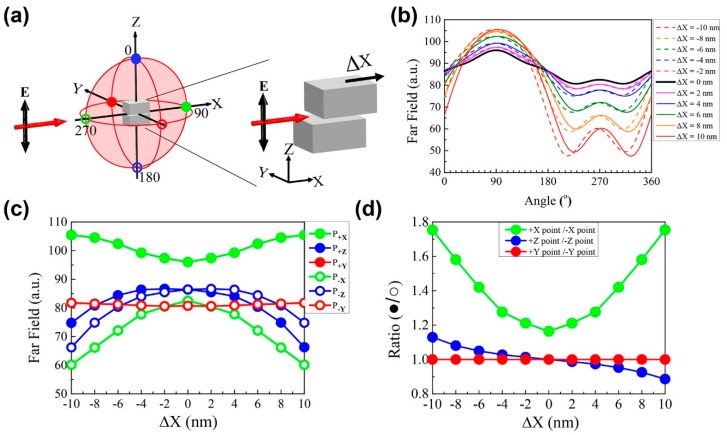
(**a**) Representation of the numerical calculation domain of the far-field region in the case when the upper block is shifted along +X direction (right side of subfigure) by the XZ plane of the red sphere. The red arrow indicates the direction of the incident light along the X-axis with the vertical electric field (E_Z_) indicated by the black arrow. (**b**) Plots of far-field intensity variations of a function of angle in the XZ plane based on the shift of the upper block. (**c**) Plots of far-field values at specific spots along the ±X, ±Y, ±Z directions. (**d**) Plots of scattering intensity ratio obtained by dividing the value at each positive axis location with the corresponding value at each negative axis location in the XZ plane.

The determination of the position of the shift of the upper block along the X direction is similar to the determination of the shift along the Y direction position based on significant changes of the scattering field. The only difference is the use of another plasmonic cavity mode that implies mode 2 is excited by the incident light with a wavelength of 1100 nm that propagates along the X direction. As previously noted in Figure 1, there is a significant change of the far-field pattern for horizontal displacements in the X-direction in the XZ plane for mode 2, as indicated by the filled-red circles in Figure 3a. We investigated the spectra of the scattering field for the shifted upper block along the X-axis in the XZ plane for displacements in the range of −10 nm to 10 nm at 2 nm interval steps, for identical conditions to those used for the determination of the results of Figure 2. In the XZ plane, the angle measurement started from the +Z-axis (0°) and also increased clockwise. Accordingly, the +X-axis was at 90°, the −Z-axis was at 180° and the −X-axis was at 270°. The dependence of the scattering intensities on the azimuthal angle is shown in Figure 3b. For the distribution analysis of the scattering intensity as a function of the angle, the observed behavior for a shift of the upper block in the X direction was analogous to that observed for a shift in the Y direction. The intensity of both the forward scattering fields (+X-axis, 90° in the XZ plane and +Y-axis, 0° in the YZ plane) were increased, while backward scattering (−X-axis, 270° in XZ plane and −Y-axis, 180 in the YZ plane) was decreased compared to the intensity of the original block (0 nm). In addition, the scattering intensity associated with the X-shift of the upper block at +Z and −Z also elicited the same response for the Y-shift.

## 4. Expansion to 2D Locations Using Scattering Ratio Maps

**Figure 4:**
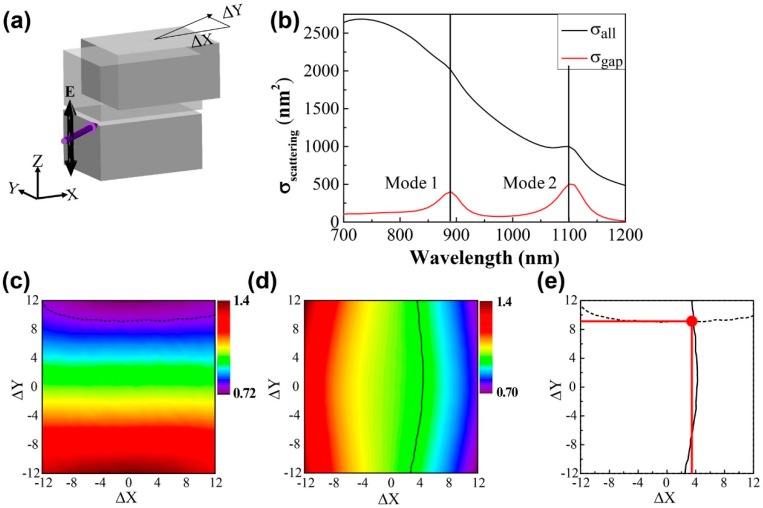
(**a**) Schematic of the movement of the upper block in the horizontal plane, XY. The purple arrow indicates the direction of the incident light propagation with respect to the blocks at an angle of 40 °. (**b**) Scattering near-field spectra of all the structures (black line) and the air gap (red line) at an incident light angle of 40°. (**c**,**d**) Plots of the scattering field intensities following horizontal and vertical shifts of ΔX = 3.7 nm and ΔY = 9.7 nm, respectively, for mode 1 (or mode 2). (**e**) Mapping plots showing the combination of the two other modes at the specific shifts of ΔX = 3.7 nm and ΔY = 9.7 nm, as represented by the dotted line and the solid lines.

Based on Figure 2 and Figure 3, the scattering intensities of the incident light at the two different wavelengths of 890 nm, and 1100 nm studied herein—which corresponding to modes 1 and 2—allows us to independently estimate the Y- or X-shifts of the upper block. Herein, mode 1 (or mode 2) was excited by the incident light that propagated along the Y direction (or X direction). In order to determine the horizontal position of the upper block for simultaneous X- and Y-shifts, we set the angle of incident light to 40°, such that 0° is assumed to be on the Y-axis, as shown in Figure 4a. For light with a wavelength in the range of 700 nm to 1200 nm, the scattering cross-sections of all the structures (black line) at an angle of 40° shows two peaks at 890 nm (mode 1), and 1100 nm (mode 2) in Figure 4b. Two distinct peaks are observed for the scattering cross section inside the gap between the two nano-blocks. In order to investigate changes in the scattering intensities, we calculated the ratio of the scattering light along the +Z- and −Z axes for a horizontal shift of the upper block with a scan step of 1 nm. 

The ratios (the scattering intensity along the +Z direction)/(the scattering intensity along the −Z direction) are plotted in Figure 4c,d as 2D maps, as functions of the X- (ΔX) and the Y-shifts (ΔY) of the upper block for the wavelengths of 890 nm and 1100 nm, respectively. In the 890 nm mapping, the ratio changed significantly along the Y direction, as shown in Figure 2. Conversely, for the 1100 nm mapping, the ratio changed primarily along the X direction. To identify the horizontal position of the upper block, the scattering intensities along the +Z and −Z axes were measured for the two wavelengths used herein, namely, 890 nm and 1100 nm, and the ratios were plotted as equi-wavelength lines in the two 2D mappings. The intersection of the two lines thus determines the horizontal position of the upper block. For example, we assumed that the horizontal position of the upper block was ΔX = 3.7 nm and ΔY = 9.7 nm. In this case, the scattering intensity ratios for 890 nm and 1100 nm were estimated and equaled 0.779 and 0.957, respectively. In the mapping shown in Figure 4c,d, the loci with values of 0.779 and 0.957 are plotted as black dashed lines and black solid lines, respectively. In Figure 4e, the two ratio lines overlap and intersect at a specific position. The horizontal position of the upper block was determined to be ΔX = 3.68 nm and ΔY = 9.02 nm. The respective discrepancies of 0.02 nm and 0.68 nm for the X- and Y-values are attributed to the results of the 1 nm spatial scan step of the theoretically prepared 2D map, which can be improved at smaller simulation resolutions. 

## 5. Conclusions

The proposed two-metal block structure enabled the determination of the horizontal displacement of one of the blocks at a nanometer resolution simply by measuring the scattering intensities of the two wavelengths. We investigated the complete range of angularly distributed scattering light in the far-field for the two orthogonal plasmon modes, and identified distinguishable scattering tendencies which had predominant features depending on the X or Y directional spatial shifts of the upper block. The measurements of the scattering field intensities at the two designated angles of the two wavelengths studied herein could facilitate the discrimination of the horizontal shift of the upper block at a high resolution. The proposed method can be validated experimentally by placing two nanoblocks on top of each other, separated by a nano-scaled air gap, and by attaching them to two atomic force microscope (AFM) tips similarly to shown in [29].

Our horizontal plasmonic ruler elicited several advantages. These included the utilization of a simple nanostructure which consisted of only two blocks and two scattering light intensities in the far-field regime that facilitated the measurements of the scattering spectrum data. In addition, because only two spots were used, the technique is relatively straightforward. Given that precision alignment techniques are extensively used in many fields, our technique is envisaged to have a wide range of practical applications. The proposed method can be used for structural analyses by monitoring binding events between molecules ranging from ions to DNA without the use of labels, or for the development of high-resolution alignment systems in conventional photolithography.
